# Real-World Data Mining for Signal Detection of Antipsychotics-Associated Adverse Events Using the Korea Adverse Event Reporting System (KAERS) Database

**DOI:** 10.3390/medicina60101714

**Published:** 2024-10-18

**Authors:** Suhyeon Moon, Minjung Ko, Yeo-Jin Choi, Sooyoung Shin

**Affiliations:** 1 Department of Biohealth Regulatory Science, Graduate School, Ajou University, Suwon 16499, Republic of Korea; suhyeun28@ajou.ac.kr (S.M.);; 2 Department of Pharmacy, College of Pharmacy, Kyung Hee University, Seoul 02447, Republic of Korea; 3 Department of Pharmacy, College of Pharmacy, Ajou University, Suwon 16499, Republic of Korea

**Keywords:** antipsychotics, drug-induced movement disorders, adverse drug events, pharmacovigilance, disproportionality analysis, signal detection analysis, real-world data, KAERS database

## Abstract

*Background and Objectives:* Recent studies suggest that the binary categorization of first-generation antipsychotics (FGAs) as being primarily responsible for extrapyramidal symptoms (EPSs) and second-generation antipsychotics (SGAs) for cardiometabolic abnormalities is an oversimplification. SGAs also demonstrate antagonistic affinity for D2 receptors, indicating their potential to induce EPSs. This study utilized the Korea Adverse Event Reporting System (KAERS) database to explore adverse drug event (ADE) signals related to both FGAs and SGAs. *Materials and Methods:* Relevant ADE reports from January 2013 to December 2022 were extracted from the KAERS database and analyzed using disproportionality analysis, employing the proportional reporting ratio (PRR), reporting odds ratio (ROR), and information component (IC) with its 95% lower confidence interval (LCI) indices. *Results:* Of the initial dataset of 2,890,702 ADE reports, those with insufficient data and duplicates were removed, resulting in a final dataset of 5249 reports for analysis. Aripiprazole, an SGA, showed signals for movement disorders, including EPSs (PRR 4.7, ROR 4.8, IC 2.2), tremors (PRR 5.3, ROR 5.4, IC 2.4), and akathisia (PRR 18.6, ROR 19.3, IC 3.5). Notably, for quetiapine, cardiovascular signals were detected, including increased blood pressure (PRR 2.1, ROR 2.3, IC 0.5), and tachyarrhythmia (PRR 13.9, ROR 14.1, IC 1.8), along with peripheral edema (PRR 2.5, ROR 2.5, IC 0.2). Metabolic abnormalities, such as weight gain and increased appetite, were identified for four SGAs: aripiprazole, olanzapine, quetiapine, and risperidone. Safety signals related to movement disorders were not detectable for FGAs, likely due to the limited number of ADE reports available for analysis. *Conclusions:* Our study findings support that the distribution of ADEs between FGAs and SGAs is not strictly binary. Aripiprazole, despite being an SGA, showed signals for extrapyramidal movement disorders. Four SGAs (aripiprazole, olanzapine, quetiapine, and risperidone) were linked to metabolic side effects, while quetiapine was associated with cardiovascular safety signals.

## 1. Introduction

Antipsychotics are the primary treatment for schizophrenia and are often used as adjunct therapy for bipolar disorder, acute psychosis, and depressive disorder [1]. First-generation antipsychotics (FGAs), such as chlorpromazine and haloperidol, improve psychotic symptoms by blocking dopamine D2 receptors and thereby reducing dopamine neurotransmission in the brain [2]. While FGAs are effective in treating the positive symptoms of schizophrenia, their use has decreased due to a lack of efficacy on negative symptoms and the serious side effect of extrapyramidal symptoms (EPSs) [1]. A variety of drug-induced movement disorders have been identified within the EPS spectrum, ranging from acute manifestations, such as akathisia, dystonia, and parkinsonism, to more chronic conditions like tardive dyskinesia [1]. These are well-known adverse effects associated with antipsychotic medications that involve dopamine-receptor blocking properties [1].

The foundation of the dopamine hypothesis of schizophrenia lies in the correlation between antidopaminergic activity, antipsychotic effects, and EPSs resulting from dopamine depletion in the extrapyramidal system [3,4]. However, this hypothesis became outdated with the advent of second-generation antipsychotics (SGAs). These newer agents, including risperidone and olanzapine, were developed to overcome the limitations of FGAs, and modeled after clozapine, the first SGA. The introduction of clozapine, the first SGA, showed promise because it effectively treated refractory schizophrenia without causing an EPS, which had previously been considered an inevitable aspect of the neuroleptic effect of antipsychotics [5,6]. However, clozapine use has been restricted by stringent regulations concerning indications and the necessity for monitoring white blood cell counts because of the risk of agranulocytosis [7]. Hence, subsequent SGAs were developed with the goal of achieving the comparable efficacy of clozapine while offering a safer side effect profile [1,8]. SGAs became the preferred treatment for schizophrenia, despite their higher cost and mixed evidence of superior efficacy compared to FGAs [9,10].

However, SGAs have fallen short of initial expectations, as they all, except clozapine, demonstrate antagonistic affinity for D2 receptors and have the potential to induce varying degrees of EPSs [3,11]. Previous meta-analyses indicated that SGAs did not offer any advantage in terms of tolerability and effectiveness compared to FGAs [11]. Additionally, post-marketing surveillance of SGAs has uncovered further adverse effects, such as metabolic side effects [1]. Recent studies have also suggested that FGAs can induce metabolic side effects, and there is no confirmed evidence supporting a higher cardiometabolic risk with SGAs relative to FGAs [12]. Consequently, the common clinical categorization of FGAs as primarily linked to EPSs and SGAs to metabolic side effects is an oversimplification and not substantiated by recent study findings [2,13].

Antipsychotic-induced adverse events, including movement disorders and cardiometabolic complications, can negatively affect patients’ quality of life by interfering with daily activities and leading to therapy nonadherence and discontinuation [14]. This nonadherence increases the risk of disease relapse and hospitalization, underscoring the need for careful management and side effect monitoring of these medications [14]. This study aims to apply data mining techniques to comprehensively assess adverse events associated with antipsychotics and detect any safety signals using adverse drug event (ADE) reports obtained from the Korea Adverse Event Reporting System (KAERS) database, and to identify specific agents associated with drug-induced adverse events which were not considered significant at the time of their marketing authorization.

## 2. Materials and Methods

### 2.1. Study Design and Data Collection

This cross-sectional study was conducted according to the Strengthening the Reporting of Observational Studies in Epidemiology (STROBE) guidelines [15]. The prespecified inclusion criteria are as follows: ADE reports associated with antipsychotics (both FGAs and SGAs) and antidopaminergic agents with known EPS risks, such as metoclopramide (a positive control), to construct a comprehensive dataset. The exclusion criteria are as follows: reports with masked codes, those with missing values, and duplicate reports. Our dataset covered the period from 1 January 2013 to 31 December 2022 as the KAERS data provided for research purposes are only available within a 10-year time frame. Therefore, the 2013–2022 period offers the most up-to-date insights into antipsychotic-associated adverse events. The KAERS is an online system developed to effectively manage and monitor reports of adverse events related to post-marketing drugs. It plays a crucial role in the collection, organization, and analysis of nationwide ADE data. The database includes details such as the type of reporter, patient demographics (including sex and age), and the suspected drug substances involved. Drugs were coded using the Anatomical Therapeutic Chemical (ATC) Classification System, and adverse events were coded according to the World Health Organization Adverse Reactions Terminology (WHO-ART). In this study, ADE reports were analyzed using the preferred terms (PT) from the WHO-ART classification, with a focus on adverse events listed on the drug’s label warnings at the time of market approval and in subsequent reports.

### 2.2. Study Medications

Antipsychotic drugs, both FGAs and SGAs, were identified in accordance with their label indications approved by the Ministry of Food and Drug Safety (MFDS). Drugs with less than two marketed products were excluded from the analysis due to the KAERS database guidelines. Additionally, antiemetic drugs, such as metoclopramide, which is known for its predominant antidopaminergic effects and its strong association with EPS risk, were included as a positive control to validate the data analysis methods. Positive controls are used to ensure the reliability of the results and to confirm that the analysis methods work as expected without affecting the overall outcome of the research [16,17].

### 2.3. Data Acquisition and Definition of Adverse Drug Events

ADEs in this study were defined as any unintended, harmful events associated with the use of pharmaceutical agents. ADEs were categorized based on causality assessments as certain, probable/likely, or possible, according to the World Health Organization Uppsala Monitoring Centre (WHO-UMC) criteria. Serious ADEs were defined as those leading to initial or extended hospitalization, permanent harm, disability, life-threatening conditions, death, or other significant medical events. All ADE reports related to antipsychotics, including both FGAs and SGAs, were collected from the KAERS database. The analysis included those ADE cases reported by various entities, including healthcare professionals, pharmaceutical companies, regional pharmacovigilance centers, and others, such as distributors and other organizations. Data extraction was conducted in February 2024; extracted details from the database included patient demographics (age and sex), medication information, patient medical histories, causality assessments, and the seriousness of the reported incident. The study protocol received approval from the Korean Institute of Drug Safety and Risk Management (Ministry of Food and Drug Safety) (No. 2312A0006) and from the institutional review board (IRB) of Ajou University (No. 202401-HB-EX-003). Informed consent was waived due to this study’s retrospective observational design based on anonymized data.

### 2.4. Statistical Analysis

Disproportionality analyses were carried out to investigate the association between antipsychotic agents and specific types of ADEs using the three key metrics: the proportional reporting ratio (PRR), the reporting odds ratio (ROR), and the information component (IC) from the Bayesian Confidence Propagation Neural Network (BCPNN) [18,19]. RORs were estimated with 95% confidence intervals (CIs). Medications with at least three reported ADE cases were included in the disproportionality analysis due to the substantially small number of ADE cases for individual agents. The criteria for signal detection were as follows: (1) PRR: PRP ≥ 2, Chi-square test statistics ≥ 4, and at least three ADE reports; (2) ROR: ROR ≥ 2, Chi-square test statistics ≥ 4, and at least three ADEs reports; and (3) IC: the lower limit of the 95% CI (95% LCI) ≥ 0 (Table 1) [18,19,20]. The ADEs that were significantly indicated by all three indices for a given drug were considered definitive pharmacovigilance signals. This approach aligns with contemporary pharmacovigilance practices and leverages statistical rigor to ensure the reliability of the findings. We used metoclopramide as a positive control to validate our analysis results. Descriptive statistics were used to analyze patient demographic information and the frequency of ADEs. All statistical analyses and data processing were conducted using SAS software (Version 9.4, SAS Institute Inc., Cary, NC, USA). For visual representation of the analytical results, heatmaps were generated using Python (Version 3.8).

## 3. Results

### 3.1. Data Filtering Process

**Figure 1:**
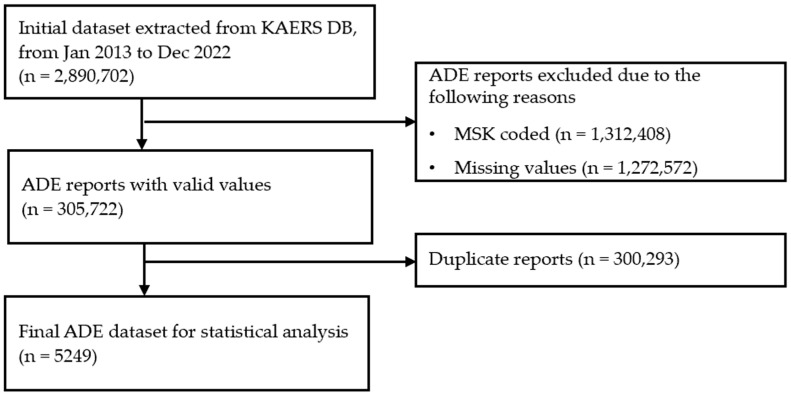
Data filtering process for the KAERS database. Abbreviations: KAERS DB, Korea Adverse Event Reporting System Database; ADE, adverse drug event; MSK, masking.

The initial dataset extracted from the KAERS database included 2,890,702 reports. To ensure data quality and reliability for analysis, several processing steps were performed, as shown in Figure 1. The first step involved removing rows associated with the “MSK” code (masking), which is used when there are two or fewer manufacturing companies, to eliminate potential bias from underrepresented products. Next, we excluded evaluation results with missing values to preserve the dataset’s integrity. After these steps, 305,722 reports remained. To avoid duplication, only the last reports from a series were included, resulting in a final dataset of 5249 reports used for signal detection analysis.

### 3.2. Baseline Demographic Information of ADE Reports

Table 2 presents the demographic data of the 5249 ADE reports pertaining to various antipsychotic medications. A demographic analysis of ADE reports revealed that 2871 (57.5%) cases involved male patients, while 2126 (42.5%) involved female patients. The age analysis showed that the 40- to 59-year age group constituted the largest proportion, with 1940 cases (46.3%), followed by the 60- to 79-year age group with 1089 cases (26.0%), the 20- to 39-year age group with 1050 (25.0%), and the under-20 age group with 83 cases (2.0%). In the causality analysis, 74 cases (1.4%) were classified as certain, 100 (1.9%) as probable/likely, and 2656 (50.6%) as possible. In terms of reporter types, pharmaceutical companies submitted 4341 reports (82.7%), followed by regional pharmacovigilance centers with 893 reports (17.0%). Regarding the types of reports, 3834 (73.0%) came from studies or research, while 1071 (20.4%) were voluntary reports. As for the final disposition of patients, 3435 patients (65.4%) recovered, 793 (15.1%) did not recover, 75 (1.4%) recovered with sequelae, and the outcome was unknown in 946 (18.0%) cases.

### 3.3. Signal Detection through Data Mining Methods

Our analysis included ADE reports covering a range of conditions, from movement disorders to metabolic abnormalities (e.g., increased appetite, weight gain, and hyperglycemia), as well as other cardiovascular adverse effects. This comprehensive dataset enabled a more extensive examination and offered real-world insights into the safety profiles of these drugs. The safety signal detection analysis revealed that only aripiprazole, olanzapine, quetiapine, and risperidone exhibited a high frequency of ADE reports associated with statistical significance, and their results are summarized in Table 3, Table 4, Table 5 and Table 6, respectively. For validation, we performed an additional signal detection analysis on metoclopramide (control). A signal for an EPS was detected with metoclopramide (PRR 25.39, ROR 27.72, IC 95% LCI 2.70), which provides compelling evidence supporting the validity of the study data. More detailed results for metoclopramide are provided in Table S1.

#### 3.3.1. Second-Generation Antipsychotic: Aripiprazole

**Figure 2:**
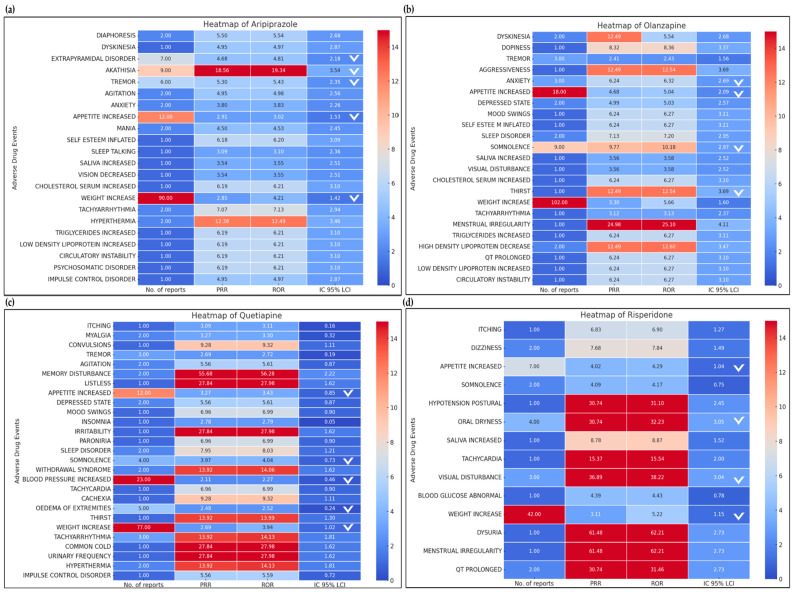
Heatmap of signal detection for antipsychotic-associated ADEs: (**a**) ADEs for aripiprazole; (**b**) ADEs for olanzapine; (**c**) ADEs for quetiapine; (**d**) ADEs for risperidone. Notes: V denotes a safety signal detected by the criteria for disproportionality analyses. Abbreviations: ADE, adverse drug event.

Table 3 shows the distribution of ADE reports along with the safety signal detection results for aripiprazole, which are also visualized in a heatmap (Figure 2a). Aripiprazole is classified as an SGA; however, significant signals were detected for movement disorders, including EPSs (PRR 4.68, ROR 4.81, IC 95% LCI 2.18), akathisia (PRR 18.56, ROR 19.34, IC 95% LCI 3.54), and tremors (PRR 5.30, ROR 5.43, IC 95% LCI 2.35). Additionally, aripiprazole showed signals for metabolic side effects, such as increased appetite (PRR 2.91, ROR 3.02, IC 95% LCI 1.53) and weight gain (PRR 2.84, ROR 4.21, IC 95% LCI 1.41). The most frequently reported ADEs were weight gain (61.2%), while EPS-related events accounted for 4.8%.

#### 3.3.2. Second-Generation Antipsychotic: Olanzapine

The distribution of ADE reports and safety signals detected for olanzapine are provided in Table 4. An overview of disproportionality analysis results is also visually summarized in a heatmap (Figure 2b). Like aripiprazole, olanzapine, which is also classified as an SGA, exhibited signals for metabolic abnormalities, such as increased appetite (PRR 4.68, ROR 5.04, IC 95% LCI 2.09) and weight gain (PRR 3.30, ROR 5.66, IC 95% LCI 1.60). Numerically, weight gain (65.0%) and increased appetite (11.5%) were the two most frequently reported ADEs for olanzapine. Additionally, olanzapine was associated with signals for somnolence (PRR 9.77, ROR 10.18, IC 95% LCI 2.97) and anxiety (PRR 6.24, ROR 6.32, IC 95% LCI 2.69). However, unlike aripiprazole, no signals for movement disorders were identified for olanzapine.

#### 3.3.3. Second-Generation Antipsychotic: Quetiapine

The distribution of ADE reports and safety signals detected for quetiapine are listed in Table 5. The graphical presentation of signal detection results is also provided in Figure 2c. Notably, quetiapine’s safety signals displayed distinct patterns compared to the previous SGAs, particularly with its association with several cardiovascular safety signals. Specifically, signals were detected for increased blood pressure (PRR 2.11, ROR 2.27, IC 95% LCI 0.46) and tachyarrhythmia (PRR 13.92, ROR 14.13, IC 95% LCI 1.81). Additionally, quetiapine was linked to signals for metabolic abnormalities, including weight gain (PRR 2.69, ROR 3.94, IC 95% LCI 1.02) and increased appetite (PRR 3.27, ROR 3.43, IC 95% LCI 0.85), as well as for peripheral edema (PRR 2.48, ROR 2.52, IC 95% LCI 0.24) and somnolence (PRR 3.97, ROR 4.04, IC 95% LCI 0.73). For quetiapine, the most frequently reported ADEs were cardiometabolic in nature, with weight gain (50.0%) being the most common, followed by increased blood pressure (14.9%). Unlike aripiprazole, quetiapine showed no signal for muscle-related disorders.

#### 3.3.4. Second-Generation Antipsychotic: Risperidone

The last SGA that exhibited significant safety signals was risperidone. Our analysis results for risperidone are summarized in Table 6 and Figure 2d. Like other SGAs, risperidone showed safety signals for metabolic side effects, such as weight gain (PRR 3.11, ROR 5.22, IC 95% LCI 1.15) and increased appetite (PRR 4.02, ROR 4.29, IC 95% LCI 1.04). Additional safety signals included dry mouth (PRR 30.74, ROR 32.23, IC 95% LCI 3.05) and visual disturbance (PRR 36.89, ROR 38.22, IC 95% LCI 3.04). For risperidone, the most frequently reported ADE was weight gain (60.9%), followed by increased appetite (10.1%).

## 4. Discussion

This pharmacovigilance study, utilizing the KAERS database in Korea, was designed to validate recent findings that suggest the binary classification of FGAs as mainly causing movement disorders and SGAs as primarily linked to metabolic abnormalities oversimplifies the issue. SGAs also exhibit antagonistic affinity for D2 receptors, suggesting their potential to cause extrapyramidal symptoms, and no studies have definitively shown that SGAs carry a greater risk of cardiometabolic issues compared to FGAs.

We performed signal detection analysis employing three disproportionality metrics and identified specific safety signals linked to individual antipsychotic agents. Among SGAs, four agents—aripiprazole, olanzapine, quetiapine, and risperidone—were detected for safety signals. Interestingly, aripiprazole, despite being classified as an SGA, exhibited signals for movement disorders, including EPSs (PRR 4.68, ROR 4.81, IC 95% LCI 2.18), tremors (PRR 5.30, ROR 5.43, IC 95% LCI 2.35), and akathisia (PRR 18.56, ROR 19.34, IC 95% LCI 3.54), which are typically known as the side effects of FGAs due to their antidopaminergic properties. Notably, cardiovascular safety signals were identified only for quetiapine, including increased blood pressure (PRR 2.11, ROR 2.27, IC 95% LCI 0.46) and tachyarrhythmia (PRR 13.92, ROR 14.13, IC 95% LCI 1.81), along with peripheral edema (PRR 2.5, ROR 2.5, IC 95% LCI 0.2). Metabolic abnormalities, such as weight gain and increased appetite, were identified solely for the aforementioned four SGAs, which were consistent with the typical side effect profiles of SGAs. This study supports the recent findings that the distribution of ADEs between FGAs and SGAs is not strictly binary, while confirming that the above four SGAs are indeed linked to metabolic abnormalities.

FGAs are typically associated with muscle-related adverse effects, such as EPSs. However, in this analysis, no signals were detected for FGAs, likely due to the limited number of ADE reports. This may be attributable to real-world antipsychotic usage patterns, where the use of FGAs has declined while the use of SGAs has increased over the past few decades. In recent years, SGAs have been more frequently prescribed due to the perception of fewer side effects compared to FGAs, particularly in the treatment of schizophrenia and other psychiatric disorders [21,22]. Additionally, haloperidol, a commonly administered antipsychotic for hospitalized patients with mental disorders, is often given via intramuscular injection rather than orally [23]. Since it is predominantly used in hospital settings, its overall usage volume is likely lower compared to that of oral SGAs, which are more commonly prescribed in outpatient settings. The constant monitoring of hospitalized patients may also contribute to the lower reporting of adverse effects among FGA-treated patients [24]. In this study, we conducted additional signal detection analyses on metoclopramide, which is known for its EPS risks due to its antidopaminergic properties, for validation purposes. For metoclopramide, signals were detected for EPSs, agitation, and decreased neutrophil count. These results are consistent with previous research and provide important evidence supporting the reliability of the data in this study [10].

Prior research has demonstrated that the adverse effects of FGAs and SGAs are frequently classified in a dichotomous manner. Muscle-related disorders, such as EPSs, have been primarily associated with a higher risk of side effects from FGAs [21]; whereas SGAs are thought to carry a lower risk of causing an EPS, but are linked to metabolic disorders and cardiovascular side effects, including diabetes and weight gain [22,25,26]. This classification is based on the understanding that the side effects of FGAs and SGAs stem from different underlying mechanisms and have been widely accepted in clinical practice for a considerable period. However, this study presents results that challenge the traditional binary perspective. Our analysis found that aripiprazole, an SGA, was associated with signals for muscle-related disorders, while no such signals were detected for FGAs. These findings suggest that SGAs can also induce muscle disorders under certain conditions, highlighting an important contrast with previous research [27,28]. Recent studies have also reported that the use of SGAs may be associated with drug-induced muscle disorders [29,30].

### Limitations

This study has several limitations. First, the results should be interpreted with caution. The KAERS database, being a spontaneous and voluntary ADE reporting system, is susceptible to biases, such as underreporting and selective reporting. Although the majority of ADE cases in this study were reported by healthcare professionals, there remains a potential for reporting bias due to differences in their interest and motivation in sharing data on antipsychotic-induced adverse events. Furthermore, drug-induced EPSs may be underreported or overlooked in real-world settings due to the multifactorial nature of movement disorders, particularly in the presence of psychiatric diseases. Patients may fail to notice symptoms of movement disorders induced by medications in their everyday lives, contributing to a lower number of reported ADE cases despite extended follow-up periods. These factors could lead to incomplete or biased data, limiting the generalizability of the findings and the ability to determine a clear causal relationship between antipsychotics, both FGAs and SGAs, and adverse events, especially movement disorders.

Additionally, as a spontaneous pharmacovigilance system, the KAERS database provided limited demographic details, including age, comorbidities, and the use of concomitant medications. This limitation may have diminished the observed impact of aging and polypharmacy on severe drug-induced movement disorder risks, leading to wider confidence intervals. Therefore, further research incorporating comprehensive patient factors is necessary to improve the understanding of drug-induced movement disorders and to optimize patient outcomes.

Despite these limitations, this study holds clinical relevance as it offers real-world evidence on the risk of drug-induced movement disorders and metabolic abnormalities associated with specific SGAs which have been under-evaluated thus far, fostering further studies and raising awareness among clinicians. Nonetheless, there is an urgent need for large-scale pharmacovigilance studies on antipsychotic-induced movement disorders and metabolic dysfunctions, including risk stratification based on comorbidities and medication types, to enhance patient care.

## 5. Conclusions

Our data mining study for ADE signal detection related to antipsychotics, based on the Korean pharmacovigilance database, supports recent findings that the distribution of ADEs between FGAs and SGAs is not strictly binary. Despite being an SGA, aripiprazole showed signals for extrapyramidal movement disorders, which are typically considered to be ADEs of FGAs. Meanwhile, among all SGAs, four SGAs (aripiprazole, olanzapine, quetiapine, and risperidone) were linked to metabolic side effects, and only quetiapine was associated with cardiovascular safety signals, consistent with the traditional binary ADE classification by generation. Further research incorporating patient factors, such as comorbidities and comedications, into ADE analysis is needed to better establish the differential ADE profiles of FGAs and SGAs.

## Figures and Tables

**Table 1:** Definition and signal detection criteria of data-mining indices.

Signal Index	Definition	Criteria of Signal
PRR	$\frac{A/(A+B)}{C/(C+D)}$	PRR ≥ 2, χ^2^ ≥ 4, A ≥ 3
ROR	$\frac{A/B}{C/D}$	ROR ≥ 2, χ^2^ ≥ 4, A ≥ 3
IC	$\log\frac{P(AE,\;drug)}{P\left(AE\right)\times P(drug)}$	Lower limit of 95% CI ≥ 0
	Specific adverse event	All other adverse events
Specific drug	A	B
All other drugs	C	D

Abbreviations: PRR, Proportional Reporting Ratio; ROR, Reporting Odds Ratio; IC, Information Component from Bayesian Confidence Propagation Neural Network; P(AE), probability of adverse event occurring; P(drug), probability of drug use in the database; CI, confidence interval; χ^2^, Chi-square.

**Table 2:** Demographic information of ADE reports.

Sex (n = 4997)	
Men	2871 (57.5%)
Women	2126 (42.5%)
Age in years (n = 4192)	
<20	83 (2.0%)
20–39	1050 (25.0%)
40–59	1940 (46.3%)
60–79	1089 (26.0%)
80–99	30 (0.7%)
Causality (n = 2830)	
Certain	74 (1.4%)
Probable/likely	100 (1.9%)
Possible	2656 (50.6%)
Reporter type (n = 5249)	
Pharmaceutical company	4341 (82.7%)
Regional pharmacovigilance center	893 (17.0%)
Medical professional	13 (0.2%)
Others (e.g., distributors or other organizations)	2 (0.0%)
Report type (n = 5249)	
Report from study/research	3834 (73.0%)
Voluntary report	1071 (20.4%)
Others	344 (6.6%)
Final disposition of patients (n = 5249)	
Recovered	3435 (65.4%)
Not recovered	793 (15.1%)
Recovered with sequelae	75 (1.4%)
Unknown	946 (18.0%)
Serious ADE	
Hospitalization	129/5249 (2.5%)
Significant medical situation	114/5249 (2.2%)

Abbreviations: ADE, adverse drug event.

**Table 3:** Signal detection analysis for aripiprazole.

Adverse Event	No. of Reports	PRR	ROR	IC 95% LCI	MFDS	FDA
Diaphoresis	2	5.50	5.54	2.68	Y	Y
Dyskinesia	1	4.95	4.97	2.87	Y	Y
Extrapyramidal disorder *	7	4.68 *	4.81 *	2.18 *	Y	Y
Akathisia *	9	18.56 *	19.34 *	3.54 *	Y	Y
Tremor *	6	5.30 *	5.43 *	2.35 *	Y	Y
Agitation	2	4.95	4.98	2.56	Y	Y
Anxiety	2	3.80	3.83	2.26	Y	Y
Appetite increased *	12	2.91 *	3.02 *	1.53 *	Y	Y
Mania	2	4.50	4.53	2.45	Y	Y
Self-esteem inflated	1	6.18	6.2	3.09	Y	Y
Sleep talking	1	3.09	3.10	2.35	Y	Y
Saliva increased	1	3.53	3.54	2.51	Y	Y
Vision decreased	1	3.53	3.54	2.51	Y	Y
Cholesterol serum increased	1	6.18	6.2	3.09	Y	Y
Weight increased *	90	2.84 *	4.21 *	1.41 *	Y	Y
Tachyarrhythmia	2	7.07	7.13	2.94	Y	Y
Hyperthermia	2	12.37	12.48	3.45	Y	Y
Triglyceride increased	1	6.18	6.21	3.09	Y	Y
Low-density lipoprotein increased	1	6.18	6.21	3.09	Y	Y
Circulatory instability	1	6.18	6.21	3.09	Y	Y
Psychosomatic disorder	1	6.18	6.21	3.09	Y	Y
Impulse control disorder	1	4.95	4.97	2.87	Y	Y

Notes: * denotes a significant result according to the criteria for signal detection analyses; Y denotes the adverse event listed on individual drug labels. Abbreviations: PRR, proportional reporting ratio; ROR, reporting odds ratio; IC, information component; LCI, lower confidence interval; MFDS, Ministry of Food and Drug Safety in Korea; FDA, Food and Drug Administration.

**Table 4:** Signal detection analysis for olanzapine.

Adverse Event	No. of Reports	PRR	ROR	IC 95% LCI	MFDS	FDA
Dyskinesia	2	12.49	5.54	2.68	Y	Y
Dopiness	1	8.32	8.36	3.37	Y	Y
Tremor	3	2.41	2.43	1.56	Y	Y
Aggressiveness	1	12.49	12.54	3.69	Y	N
Anxiety *	3	6.24 *	6.32 *	2.69 *	Y	Y
Appetite increased *	18	4.68 *	5.04 *	2.09 *	Y	Y
Depressed state	2	4.99	5.03	2.57	Y	Y
Mood swings	1	6.24	6.27	3.11	Y	Y
Self-esteem inflated	1	6.24	6.27	3.11	N	N
Sleep disorder	2	7.13	7.2	2.95	Y	Y
Somnolence *	9	9.77 *	10.18 *	2.97 *	Y	Y
Saliva increased	1	3.56	3.58	2.52	Y	Y
Visual disturbance	1	3.56	3.58	2.52	Y	Y
Cholesterol serum increased	1	6.24	6.27	3.10	Y	Y
Thirst	1	12.49	12.54	3.69	Y	Y
Weight increased *	102	3.30 *	5.66 *	1.60 *	Y	Y
Tachyarrhythmia	1	3.12	3.13	2.37	Y	Y
Menstrual irregularity	1	24.98	25.10	4.11	Y	Y
Triglyceride increased	1	6.24	6.27	3.11	Y	Y
High-density lipoprotein decreased	1	6.24	6.27	3.11	Y	Y
QT prolonged	2	12.49	12.60	3.47	Y	Y
Low-density lipoprotein increased	1	6.24	6.27	3.10	Y	Y
Circulatory instability	1	6.24	6.27	3.10	N	N

Notes: * denotes a significant result according to the criteria for signal detection analyses; Y denotes the adverse event listed on individual drug labels; N denotes the adverse event not listed on individual drug labels. Abbreviations: PRR, proportional reporting ratio; ROR, reporting odds ratio; IC, information component; LCI, lower confidence interval; MFDS, Ministry of Food and Drug Safety in Korea; FDA, Food and Drug Administration.

**Table 5:** Signal detection analysis for quetiapine.

Adverse Event	No. of Reports	PRR	ROR	IC 95% LCI	MFDS	FDA
Itching	1	3.09	3.11	0.16	N	N
Myalgia	2	3.27	3.30	0.32	Y	Y
Convulsions	1	9.28	9.32	1.11	Y	N
Tremor	3	2.69	2.72	0.19	Y	Y
Agitation	2	5.56	5.61	0.87	Y	Y
Memory disturbance	2	55.68	56.28	2.22	N	Y
Listless	1	27.84	27.98	1.62	Y	Y
Appetite increased *	12	3.27 *	3.43 *	0.85 *	Y	Y
Depressed state	2	5.56	5.61	0.87	Y	Y
Mood swings	1	6.96	6.99	0.90	Y	Y
Insomnia	1	2.78	2.79	0.05	Y	Y
Irritability	1	27.84	27.98	1.62	Y	Y
Paroniria	1	6.96	6.99	0.90	Y	Y
Sleep disorder	2	7.95	8.03	1.21	Y	Y
Somnolence *	4	3.97 *	4.04 *	0.73 *	Y	Y
Withdrawal syndrome	2	13.92	14.06	1.62	Y	Y
Blood pressure increased *	23	2.11 *	2.27 *	0.46 *	Y	Y
Tachycardia	1	6.96	6.99	0.90	Y	Y
Cachexia	1	9.28	9.32	1.11	N	N
Oedema of extremities *	5	2.48 *	2.52 *	0.24 *	Y	Y
Thirst	1	13.92	13.99	1.3	Y	Y
Weight increased *	77	2.69 *	3.94 *	1.02 *	Y	Y
Tachyarrhythmia *	3	13.92 *	14.13 *	1.81 *	Y	Y
Common cold	1	27.84	27.98	1.62	N	N
Urinary frequency	1	27.84	27.98	1.62	N	N
Hyperthermia	2	13.92	14.13	1.81	N	N
Impulse control disorder	1	5.56	5.59	0.72	Y	Y

Notes: * denotes a significant result according to the criteria for signal detection analyses; Y denotes the adverse event listed on individual drug labels; N denotes the adverse event not listed on individual drug labels. Abbreviations: PRR, proportional reporting ratio; ROR, reporting odds ratio; IC, information component; LCI, lower confidence interval; MFDS, Ministry of Food and Drug Safety in Korea; FDA, Food and Drug Administration.

**Table 6:** Signal detection analysis for risperidone.

Adverse Event	No. of Reports	PRR	ROR	IC 95% LCI	MFDS	FDA
Itching	1	6.83	6.90	1.27	Y	N
Dizziness	2	7.68	7.84	1.49	Y	Y
Appetite increased *	7	4.02 *	4.29 *	1.04 *	Y	Y
Somnolence	2	4.09	4.17	0.75	Y	Y
Hypotension postural	1	30.74	31.10	2.45	Y	Y
Oral Dryness *	4	30.74 *	32.23 *	3.05 *	Y	Y
Saliva increased	1	8.78	8.87	1.52	Y	Y
Tachycardia	1	15.37	15.54	2.00	Y	Y
Visual disturbance *	3	36.89 *	38.22 *	3.04 *	Y	Y
Blood glucose abnormal	1	4.39	4.43	0.78	Y	Y
Weight increased *	42	3.11 *	5.22 *	1.15 *	Y	Y
Dysuria	1	61.48	62.21	2.73	Y	Y
Menstrual irregularity	1	61.48	62.21	2.73	Y	Y
QT Prolonged	2	30.74	31.46	2.73	Y	Y

Notes: * denotes a significant result according to the criteria for signal detection analyses; Y denotes the adverse event listed on individual drug labels; N denotes the adverse event not listed on individual drug labels. Abbreviations: PRR, proportional reporting ratio; ROR, reporting odds ratio; IC, information component; LCI, lower confidence interval; MFDS, Ministry of Food and Drug Safety in Korea; FDA, Food and Drug Administration.

## Data Availability

Data are available from the corresponding author upon reasonable request. Restrictions may apply to the availability of some of the data, which were used under permission from the Korean Institute of Drug Safety and Risk Management.

## Supplementary Materials

The following supporting information can be downloaded at: https://www.mdpi.com/article/10.3390/medicina60101714/s1, Table S1. Singal detection results for metoclopramide; Table S2. Full terms for abbreviations used in the manuscript; Table S3. Comparison of previous study findings and our study results; Table S4. Signal detection results for aripiprazole; Table S5. Signal detection results for olanzapine; Table S6. Signal detection results for quetiapine; Table S7. Signal detection results for risperidone.
