# Supplementary material for: Real-World Data Mining for Signal Detection of Antipsychotics-Associated Adverse Events Using the Korea Adverse Event Reporting System (KAERS) Database
Source: Medicina (Kaunas). 2024 Oct 18;60(10):1714. doi: 10.3390/medicina60101714 (PMC11509233; doi:10.3390/medicina60101714)
Supplement: Supplementary file 1 [file medicina-60-01714-s001.zip › medicina-3245828-supplementary.pdf]

Supplementary Materials

Table S1. Signal detection results for metoclopramide

Adverse event	No. of reports	PRR	ROR	IC 95% LCI	MFDS	FDA
Diaphoresis*	5	13.32*	13.52*	1.64*	Y	Y
Urticaria	1	5.32	5.34	0.34	Y	Y
Hoarseness*	3	3.68*	3.71*	0.39*	N	N
Extrapyramidal disorder*	27	25.39*	27.72*	2.70*	Y	Y
Confusion	1	5.32	5.34	0.34	Y	Y
Agitation*	4	7.99*	8.08*	1.18*	Y	Y
Hypotension*	7	2.54*	2.57*	0.28*	Y	Y
Tachycardia*	3	23.98*	24.2*	1.63*	Y	Y
Cachexia	1	5.32	5.34	0.34	N	N
Nose bleed	1	5.32	5.34	0.34	N	N
Dyspnea*	6	2.82*	2.85*	0.35*	Y	Y
Hypoxemia	1	5.32	5.34	0.34	N	N
Decreased neutrophil count*	13	1.71*	1.74*	0.004*	N	N
Dysuria	1	15.98	16.03	0.86	N	N
Chest discomfort	5	2.35	2.37	0.08	Y	Y
Decreased white blood cell count	2	3.99	4.01	0.32	N	N
Xerophthalmia	1	5.32	5.34	0.34	N	N
Feeling of warmth	2	10.65	10.72	1.04	N	N

Notes: * denotes a significant result according to the criteria for signal detection analyses; Y denotes the adverse event listed on individual drug labels; N denotes the adverse event not listed on individual drug labels.
Abbreviations: PRR, proportional reporting ratio; ROR, reporting odds ratio; IC, information component; LCI, lower confidence interval; MFDS, Ministry of Food and Drug Safety in Korea; FDA, Food and Drug Administration.

Table S2. Full terms for abbreviations used in the manuscript

Abbreviation	Full term
ADE	Adverse drug event
EPS	Extrapyramidal symptoms
FGA	First-generation antipsychotic
SGA	Second-generation antipsychotic
PRR	Proportional reporting ratio
ROR	Reporting odds ratio
IC	Information component
LCI	Lower confidence interval
WHO-UMC	World Health Organization-Uppsala Monitoring Centre
WHO-ART	World Health Organization Adverse Reactions Terminology
PT	Preferred term
KAERS	Korea Adverse Event Reporting System
BCPNN	Bayesian Confidence Propagation Neural Network
FDA	Food and Drug Administration
MFDS	Ministry of Food and Drug Safety
CI	Confidence interval
χ^2	Chi-square Test

Notes: All abbreviations were defined upon their first occurrence within the text.

Table S3. Comparison of previous study findings and our study results

Study	Drug class	Adverse events/ Study focus	Study findings	Our study results
Leucht et al. (2009) [9]	FGA vs. SGA	EPS	FGAs had a higher EPS risk than SGAs.	No EPS signals were detected for FGAs. Aripiprazole (SGA) was associated with signals for movement disorders, including EPS.
Misdrahi et al. (2019) [25]	Antipsychotics (General)	EPS	No specific comparative risk was assessed, but FGAs were suggested to have a higher risk for EPS than SGAs.	Aripiprazole was associated with signals for movement disorders, including EPS.
Ventura et al. (2009) [26]	FGA vs. SGA	Functional and neurocognitive impact	FGAs showed greater neurocognitive side effects than SGAs.	No neurocognitive safety signals were detected in our analysis.
Kannarkat et al. (2022) [27]	Newer Antipsychotics	Drug-induced movement disorders	SGAs were associated with drug-induced muscle disorders.	Aripiprazole was associated with signals for movement disorders, including EPS.
Tschoner et al. (2007) [21]	SGAs	Metabolic disorders	SGAs were linked to metabolic disorders.	Several SGAs were associated with signals for metabolic side effects.
Lieberman et al. (2005) [10]	FGAs vs. SGAs	Various side effects	SGAs had a safer overall side effect profile than FGAs.	No EPS signals were detected for FGAs, potentially due to their limited use especially in outpatient settings.
Miyamoto et al. (2005) [24]	Antipsychotics (General)	Mechanism of action	FGAs primarily affect dopamine pathways; SGAs have a wide range of effects.	Aripiprazole was associated with signals for movement disorders, suggesting the potential antidopaminergic properties of SGAs.

Abbreviations: FGA, first-generation antipsychotic; SGA, second-generation antipsychotic; EPS, extrapyramidal symptoms.

Table S4. Signal detection results for aripiprazole

Adverse event	n11[1)]	n12[2)]	n21[3)]	n22[4)]	PRR	ROR	IC	P-value
Extrapyramidal disorder	7	205	37	5212	4.68	4.81	2.19	< 0.001
Akathisia	9	203	12	5237	18.57	19.35	3.54	< 0.001
Tremor	6	206	28	5221	5.31	5.43	2.36	< 0.001
Increased appetite	12	200	102	5147	2.91	3.03	1.54	< 0.001
Weight gain	90	122	782	4467	2.85	4.21	1.42	< 0.001

Notes: P-values were calculated using the Chi-square test, which evaluates the independence of the observed frequencies in the 2x2 contingency table; 1) n11: Number of cases with the adverse event in patients treated with aripiprazole, 2) n12: Number of cases without the adverse event in patients treated with aripiprazole, 3) n21: Number of cases with the adverse event in patients not treated with aripiprazole, 4) n22: Number of cases without the adverse event in patients not treated with aripiprazole.

Table S5. Signal detection results for olanzapine

Adverse event	n11[1)]	n12[2)]	n21[3)]	n22[4)]	PRR	ROR	IC	P-value
Anxiety	3	199	12	5035	6.25	6.33	2.69	< 0.001
Increased appetite	18	184	96	4951	4.68	5.05	2.09	< 0.001
Somnolence	9	193	23	5024	9.78	10.19	2.97	< 0.001
Weight gain	102	100	770	4277	3.31	5.67	1.61	< 0.001
Anxiety	3	199	12	5035	6.25	6.33	2.69	0.01

Notes: P-values were calculated using the Chi-square test, which evaluates the independence of the observed frequencies in the 2x2 contingency table; 1) n11: Number of cases with the adverse event in patients treated with olanzapine, 2) n12: Number of cases without the adverse event in patients treated with olanzapine, 3) n21: Number of cases with the adverse event in patients not treated with olanzapine, 4) n22: Number of cases without the adverse event in patients not treated with olanzapine.

Table S6. Signal detection results for quetiapine

Adverse event	n11[1)]	n12[2)]	n21[3)]	n22[4)]	PRR	ROR	IC	P-value
Increased appetite	12	170	102	4965	3.27	3.43	1.69	< 0.001
Somnolence	4	178	28	5039	3.97	4.04	2.11	0.05
Increased blood pressure	23	159	303	4764	2.11	2.27	1.07	< 0.001
Edema of extremities	5	177	56	5011	2.48	2.52	1.47	0.093
Weight gain	77	105	795	4272	2.69	3.94	1.35	< 0.001
Tachyarrhythmia	3	179	6	5061	13.92	14.13	3.52	< 0.001

Notes: P-values were calculated using the Chi-square test, which evaluates the independence of the observed frequencies in the 2x2 contingency table; 1) n11: Number of cases with the adverse event in patients treated with quetiapine, 2) n12: Number of cases without the adverse event in patients treated with quetiapine, 3) n21: Number of cases with the adverse event in patients not treated with quetiapine, 4) n22: Number of cases without the adverse event in patients not treated with quetiapine.

Table S7. Signal detection results for risperidone

Adverse event	n11[1)]	n12[2)]	n21[3)]	n22[4)]	PRR	ROR	IC	P-value
Increased appetite	7	77	107	5058	4.02	4.30	2.10	< 0.001
Dry mouth	4	80	8	5157	30.74	32.23	4.57	< 0.001
Visual disturbance	3	81	5	5160	36.89	38.22	4.78	< 0.001
Weight gain	42	42	830	4335	3.11	5.22	1.60	< 0.001

Notes: P-values were calculated using the Chi-square test, which evaluates the independence of the observed frequencies in the 2x2 contingency table; 1) n11: Number of cases with the adverse event in patients treated with risperidone, 2) n12: Number of cases without the adverse event in patients treated with risperidone, 3) n21: Number of cases with the adverse event in patients not treated with risperidone, 4) n22: Number of cases without the adverse event in patients not treated with risperidone.
